# The Infant at Birth

**Published:** 1905-03-11

**Authors:** 


### THE INFANT AT BIRTH.

In a lecture given at the Institute of Hygiene last week on the Physiology, Care, and Management of the Infant at Birth, Dr. W. G. McDowell referred to the paucity of literature bearing on the subject and impressed upon his hearers the necessity of using to the best advantage such practical information as is available in order to preserve the infant from the evil influences that surround him at the present time. It is not generally allowed, he said, that most babies are born perfectly healthy, yet this is so, for Nature has not only a wonderful way of pinching out an undesirable strain, but also a tendency to adjustment in all her processes. Those who enter life burdened with hereditary taint, and those who are physically or mentally decrepit at birth are, after all, comparatively few. What then is the cause of so much mortality during the first years of life, when no less than about 15 per cent. of those born cease to exist? The mother has, crystallised into instinct, the experience of ages to guide her. Why should she err in the bringing up of her offspring? But she does, and it is this fact which calls for the admonition to go back to nature.

The child's physiological conditions should be studied and his requirements learned. The imperfect development of the brain, till the end of the seventh year, is a fact that should prevent a child from being sent to school at an early age. The children of the poorer classes are, oftener than not, sent to school at the age of three, when they should be living the freest possible outdoor life. A child's brain loves activity, but it must work in its own way—not at the behest of taskmasters who fix his attention for long periods and try to compel steady application. Dr. Jacobi has maintained that no child should commence school studies till it is nine years of age, and there is a good deal to be said in favour of his contention.

In speaking of the special senses at birth, Dr. McDowell pointed out that sight and hearing are defective to a degree that might greatly surprise the mother if she were allowed to find it out for herself in the case of her own young babe. The infant is able to detect the presence of a strong light and to differentiate between light and darkness but it cannot see its mother's face for some weeks. The recognition of colours comes only after a few months. He first distinguishes yellow, red, pure white, gray, and black, while green and blue come later. Taste is evident at birth, but hearing is not sufficiently developed for him to hear much more than his own cry.

One of the strongest points insisted upon was, of course, the avoidance of adventitious feeding soon after birth, and the advisability of depending upon the food provided by nature which is the only food physiologically fit for consumption by the new-born infant. Nature actually goes out of her way to make a special kind of milk for the infant's first few meals to suit his temporary needs, but it is an all too common thing to find Nature forestalled by ignorance and the child dosed with a confection of traditional veneration and misplaced respect—butter and sugar.

The final remark of the lecturer was to the effect that those who disregard the ways of Nature in bringing up their children, go out to meet sorrow and generally have not far to go.

### MENTAL DISORDERS OF DECAY.

Dr. Savage, in the last of his Lettsomian Lectures, dealt with the later manifestations of insanity. He divided these into :—(1) The disorders of premature decay (*i.e.* dementia præcox) ; (2) the general paralytic group ; (3) the true senile psychoses.

Of dementia præcox he said that it might be called "postponed idiocy." The younger the child affected the worse the prognosis, while if youth and a neurotic inheritance coincided in any given case the prospect was bad in the extreme. Further, the prognosis was worse in the case of a boy than of a girl. The symptoms were very variable at the onset. There might be no more than a complaint that the child was prone to act foolishly, to exhibit emotion out of normal proportion to exciting causes. In the case of young men it was common to find alternating phases of dissipation and contrition frequently repeated. Sexual perversions were often associated with cases of this kind, but it was dubious how far they stood in the light of cause and how far of effect. Sexual hypochondria, indolence, solitariness were all to be found heralding or accompanying the condition of dementia præcox.

Of the general paralytic group the lecturer was unable to speak, owing to exigencies of time. He passed on to the true senile conditions, observing that they were often dependent on disease of other parts, notably the arteries. In old men the ruling temperament of life tended to pass out of control : thus the habitually careful man became a miser ; the puritanical person came to the conclusion that his soul was irretrievably lost. This loss of control was further exemplified by the proverbial verbosity of the old, with the not uncommon recrudescence of sexual inclination. Referring to the saying that late marriage means early death the lecturer observed that in a good many cases the fact that an old man marries is evidence that he is already, so to speak, dying. His new-found youth is, in fact, the herald announcing his mental deterioration.

With loss of control goes loss of memory—of recent memory, that is to say, and with this there may concur strange revivals of by-gone events. This revival the lecturer instanced in the following case. A man well past sixty became obsessed with the idea that his wife, with whom he had lived in happiness for more than thirty years, was false to him. It appeared that the lady had, in fact, been guilty of a liaison quite early in her married life. This had, however, been forgiven, and had not been mentioned for some thirty years. Yet suddenly the memory was revived in the guise of an event of the recent past.

